# *QuickStats:* Prevalence of Obesity* and Severe Obesity^†^ Among Persons Aged 2–19 Years — National Health and Nutrition Examination Survey, 1999–2000 through 2017–2018

**DOI:** 10.15585/mmwr.mm6913a6

**Published:** 2020-04-03

**Figure:**
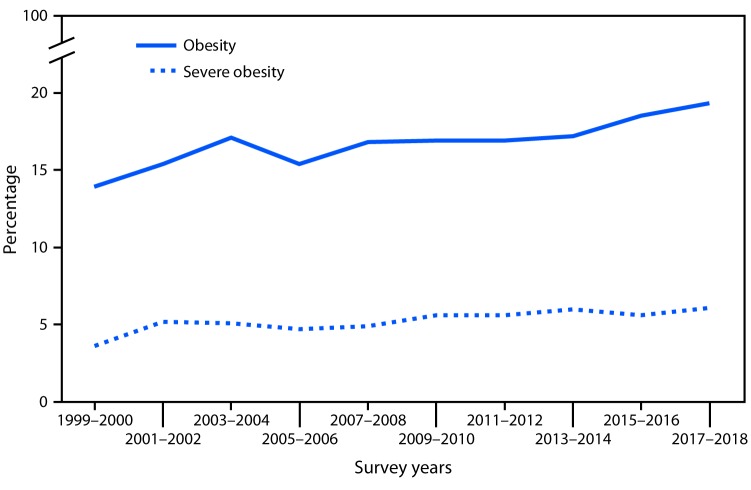
From 1999–2000 to 2017–2018, the prevalence of obesity among persons aged 2–19 years increased from 13.9% to 19.3%, and the prevalence of severe obesity increased from 3.6% to 6.1%.

**Source:** National Center for Health Statistics, National Health and Nutrition Examination Survey, 1999–2000 to 2017–2018. https://www.cdc.gov/nchs/nhanes.htm.

